# Detecting Important Risk Factors of Survival Time of Lung Cancer Patients Using Censored Quintile Regression

**DOI:** 10.31557/APJCP.2019.20.9.2583

**Published:** 2019

**Authors:** Payam Amini, Malek Abazari, Behnaz Alafchi

**Affiliations:** 1 *Department of Epidemiology and Reproductive Health, Reproductive Epidemiology Research Center, Royan Institute for Reproductive Biomedicine, ACECR, Tehran,*; 2 *Department of Public Health, School of Public Health, Ardabil University of Medical Sciences, Ardabil,*; 3 *Department of Biostatistics, School of Public Health, Hamadan University of Medical Sciences, Hamadan, Iran. *

**Keywords:** Lung cancer, censored quintile regression, survival, cohort studies

## Abstract

**Objective:**

Lung cancer is the most frequently diagnosed cancer and the most leading cause of death due to cancer worldwide. This study aimed to use censored quintile regression model to estimate the effect of potential risk factors on survival of lung cancer patients.

**Methods:**

In this study we used a dataset of a retrospective cohort study conducted in West Azerbaijan (during 2007 and 2014). Demographic variables included age, and gender and biological variables included Eastern Cooperative Oncology Group, smoking, tumor type, stage, metastasis, and treatment were investigated as risk factors of survival of lung cancer patients by using censored quintile regression.

**Result:**

The mean (± standard deviation) age of the 347 lung cancer patients was 63.48 (± 13.55) years. The survival time ranges from 11 to 91. A total of 240 (67.6%) experienced death by the end of the study. The impact of Eastern Cooperative Oncology Group (IV), smoking and treatment were significant for most of quintiles (p<0.05). Stage of cancer and metastasis are the other affective risk factors on the survival of lung cancer patients(p<0.05). It was shown that Eastern Cooperative Oncology, smoking habit and treatment were significantly associated with a shorter time-to-death progression.

**Conclusion:**

As censored quintile regression could consider time-varying effects and the interpretation of its regression coefficients are easy it could be an adequate choice for analyzing survival data.

## Introduction

Cancer is the most common chronic disease and the most important causes of death and disability, worldwide. Cancer after cardiovascular diseases is the second cause of death in the developed countries (del Pilar Díaz et al., 2009) and after cardiovascular diseases and accidents is the third cause of death in Iran (Farahmand et al., 2011). Among all types of cancer, lung cancer is the most common cancer in men and the fourth most frequent cancer in women. The highest rates of lung cancer are seen in West Asia, South Africa, and the Caribbean, among less developed countries with an age-standardized rate (ASR) of 25.7 to 32.2 per 100,000 (Ferlay et al., 2010). From a public health standpoint, development of lung cancer is so worrying because of its worst prognosis (Olsen, 1997). 

Based on classification of the America Cancer Society according to the type of cells in the lung tissue, there are two types of lung cancer including small cell carcinoma (SCC) and non-small cell carcinoma (NSCC) (Macdonald et al., 2004). The most common type of lung cancer is SCC and about 67% of the all cases which diagnosed with this type have metastatic disease because this type of disease has rapid growth and spreads to the other organs (Horton et al., 2005). There are three main subtypes of NSCC which are adenocarcinoma, squamous-cell carcinoma and large-cell carcinoma. Often NSCC patients are diagnosed in advanced stages (Ettinger et al., 2012).

Lung cancer is the most frequently diagnosed cancer and the most leading cause of death due to cancer worldwide with 18.2% of total cancer deaths (Ferlay et al., 2010; Zahir and Mirtalebi, 2012). It has been arising first in Western countries in the 1930s and its rate is rising in subsequent decades (Ridge et al., 2013). Among women the incidence of lung cancer has increased by 2.6% annually during the past decade, while the trend for men in this period has shown a reduction of 0.8% per year. Although, it has been noted that lung cancer in the age group 45–64 among women is as among as men (Barlow, 2006). Lung cancer does account for more deaths than the other cancers in both genders (Howlader et al., 2012). The overall 5-year survival rate for all stages was evaluated as 16.8% in 2004 which has slightly improved over time. The survival rate for lung cancer patients vary with stages at diagnosis (52.2% for localized disease, to 25% for regional disease, to 4% for distant disease) (Howlader et al., 2012). Unfortunately, most of the patients have diagnosed in advanced stages and only 15% of patients are diagnosed in the early localized stage (Ridge et al., 2013). Lung cancer is the second fatal cancer after gastric cancer according to Disability Adjusted Life Years Index with 56.3 per hundred thousand people (Bollschweiler et al., 2002).

Lung cancer is known as a malignant lung tumor characterized by uncontrolled cell growth in tissues of the lung (Eramo et al., 2008). One of the most important indicators for disease control and evaluation of treatments is the survival rate of patients with cancer (Kleinbaum and Klein, 2010). The epidemiological researches showed that lung cancer risk is strongly associated with tobacco, spatially cigarette, use (Hill and Doll, 1999; Ridge et al., 2013). Such that, approximately 80% of the lung cancer cases are caused by smoking that means lung cancer could be prevented successfully (Barlow, 2006). In the United States, about 90% of lung cancer deaths in men and about 80% of lung cancer deaths in women are caused by smoking (Control and Prevention, 1989; Shopland, 1995). Different studies have shown that some occupational factors like pollution, nutritional factors, and some demographic and disease related factors are other important risk factors such as age, sex, and weight loss which affect the survival time of the patients with lung cancer (Hill and Doll, 1999; Control and Prevention, 1989; Shopland, 1995; Cox, 1984). 

Time to event is the most common types of data in medical problems. Lots of statistical tools are available to analyze this data including parametric models such as Weibull distribution, semi-parametric approaches such as Cox and quantile regression, and non-parametric tools such as Kaplan-Meyer estimations (Cox and Oakes, 1984; Portnoy, 2003). Although the prevalence of lung cancer in the West Azerbaijan province is high, few studies have been done to assess the survival rate of patients. The present study intends to determine the important risk factors on the survival of patients with lung cancer using censored quantile regression (CQR) model. 

## Materials and Methods

*Data*

This retrospective cohort study includes the demographic and biological information of all patients with lung cancer referred to university hospitals of West Azerbaijan province during 2007 and 2014. Demographic variables included age at time of diagnosis, and gender. Biological variables included Eastern Cooperative Oncology Group (ECOG) performance (I, II/ III/IV), smoking habit (yes/no), tumor type (Carcinoid and others, small cell lung cancers: SCLC, non-small cell lung cancers: NSCLC), cancer stage (I, II, III/IV), metastasis (yes/no), treatment (Received / Not-received). The patients were followed by the June 2014 and their information obtained from medical records and through phone calls and home visits. The patients who lost to follow up or died for reasons other than lung cancer or were alive at the end of the study were considered as censored. The duration of survival time was considered as the difference between the time of diagnosis and the end of the study period. 

*Statistical analysis*

To show the descriptive statistics of the patients mean (± standard error) and frequency (percentage) were used for continuous and categorical variables, respectively. The mean survival time was compared between the categories of variables using log-rank test. 

Regarding the skewness in the distribution of survival time, CQR was utilized to find the overall survival of the lung cancer patients using adjusted effects of variables (Fitzenberger, 1997; Portnoy, 2003; Wang and Wang, 2009). This model provides the clinicians and the physicians with numerous quantiles of survival time based on several risk factors (Fitzenberger, 1997; Portnoy, 2003; Wang and Wang, 2009). This model estimates the pth quantile of survival time (Q_p_) as follows in which X’ is the vector of covariates and factors, β_p_ is the vector of coefficients for the p^th^ quantile.

$Q\left(p\backslash X\right)=X^{'}\beta_{p}$

**Table 1:** The Patients’ Characteristics and the Results of Log-Rank Test

Variable	n (%)	Death (% within row)	Mean survival time (month)	Log-Rank test	
		240 (67.6)	Mean (SE)	Chi-square test	p-value
Gender				0.863	0.353
Female	99 (27.9)	58 (58.6)	1387 (±1.47)		
Male	256 (72.1)	182 (71.1)	12.56 (±0.91)		
ECOG				36.701	<0.001
I, II	80 (22.5)	45 (56.3)	19.22 (±2.11)		
III	161 (45.4)	98 (60.9)	13.61 (±1.11)		
IV	110 (31)	93 (84.5)	7.87 (±0.953)		
Smoking habit				11.613	0.001
Yes	245 (69)	172 (70.2)	11.36 (±0.943)		
No	109 (30.7)	68 (62.4)	16.02 (±1.25)		
Tumor				0.818	0.664
Carcinoid & others	58 (16.3)	36 (62.1)	15.22 (±2.04)		
SCLC	27 (7.6)	21 (77.8)	16.91 (±14.25)		
NSCLC	216 (60.8)	140 (64.8)	12.32 (±1.85)		
Stage				5.126	0.024
I, II, III	100 (28.2)	58 (58.0)	15.88 (±1.89)		
IV	213 (60)	152 (71.4)	11.66 (±0.92)		
Treatment				47.122	<0.001
Yes	186 (52.4)	131 (70.4)	14.77 (±1.01)		
No	100 (28.2)	84 (84.0)	6.09 (±0.89)		
Metastasis				12.069	0.001
Yes	191 (53.8)	138 (72.3)	15.76 (±1.31)		
No	164 (46.2)	102 (62.2)	10.47 (±0.88)		

**Table 2:** The Results of Censored Quantile Regression Assessing the Effect of Sex, Age, Surgery, SES and Time Interval Symptom Diagnosis on Survival Time Quantiles

Quantiles	Intercept	Age	ECOG (III)	ECOG (IV)	Smoking (Yes)	Cancer Stage (IV)	Treat (No)	Metastasis (Yes)
5	**1.96** **(0.42)**	-0 .06 (0 .11)	-0.03 (0.25)	-0.27 (0.3)	-0.66 (0.4)	0 .08 (0.18)	-0.27 (0.14)	-0.03 (0.14)
10	**4.2** **(1.86)**	-0.01 (0.01)	-0.63 (1.06)	-1.18 (1.28)	**-1.84** **(0.79)**	0.32 (0.75)	-0.57 (0.88)	-0.47 (0.89)
15	**5.56** **(1.21)**	-0.01 (0.02)	-1.12 (0.74)	-1.25 (1.36)	**-2.28** **(0.67)**	0.12 (0.55)	**-1.06** **(0.42)**	-0.13 (1.41)
20	**6.87** **(2.38)**	-0 .01 (0.04)	-1.85 (1.81)	-1.94 (1.43)	-2.74 (1.59)	0.12 (0.61	**-1.26** **(0.5)**	-0.04 (1.3)
25	**8.76** **(2.41)**	-0.01 (0.03)	-2.06 (1.7)	-2.22 (1.33)	-3.67 (2.96)	0.56 (0.82)	**-2.03** **(0.78)**	-0.39 (0.73)
30	**10.45** **(1.64)**	-0.01 (0.02)	-2.07 (1.27)	**-2.37** **(1.22)**	**-4.63** **(1.2)**	0.86 (1.16)	**-2.49** **(1.07)**	-0.81 (1.01)
35	**11.55** **(3.82)**	-0.03 (0.06)	-2.47 (1.79)	-2.74 (1.7)	**-4.6** **(2.37)**	1.12 (1.34)	**-3.31** **(1.09)**	-1.01 (0.7)
40	**15.55** **(4.38)**	-0.02 (0.03)	-3.66 (3.78)	-4.09 (4.05)	**-6.74** **(2.72)**	0.71 (1.24)	**-3.71** **(1.77)**	-0.68 (0.88)
45	**17.79** **(5.67)**	-0.01 (0.04)	-4.36 (6.29)	-5.14 (6.51)	**-6.74** **(1.78)**	1.27 (2.75)	**-5.23** **(2.22)**	-0.92 (1.69)
50	**21.94** **(3.72v**	-0.01 (0.01)	-6.99 (4.39)	**-7.99** **(4.19)**	**-7.02** **(1.65)**	1.57 (2.1)	**-6.09** **(1.02)**	-1.34 (1.88)
55	**23.72** **(4.5)**	-0.01 (0.02)	-7.43 (4.05)	**-8.42** **(3.4)**	**-6.98** **(2.8)**	2.05 (1.94)	**-7.17** **(2.57)**	-2.01 (1.71)
60	**27.73** **(6.98)**	-0.03 (0.05)	-6.32 (4.36)	**-8.31** **(4.02)**	-9.3 (6.25)	2.03 (1.79)	**-8.84** **(4.37)**	-1.89 (1.2)
65	**29.96** **(3.63)**	-0.01 (0.05)	-4.81 (3.39)	**-7.77** **(3.26)**	**-10.53** **(3.05)**	1.56 (1.99)	**-9.64** **(1.86)**	-1.86 (0.92)
70	**31.03** **(3.71)**	-0.03 (0.05)	-5.71 (4.73)	-9.34 (6.3)	**-9.72** **(2.83)**	1.4 (2.74)	**-10.1** **(1.72)**	-1.18 (1.32)
75	**34.63** **(7.02)**	-0.04 (0.01)	-7.85 (7.08)	-11.83 (6.62)	**-10.19** **(3.13)**	1.44 (3.81)	**-10.68** **(1.38)**	-1.62 (2.21)
80	**45.19** **(13.91)**	-0.07 (0.13)	-7.98 (7.92)	-14.56 (10.4)	**-10.97** **(2.23)**	-5.08 (10.41)	**-11.16** **(3.79)**	-1.5 (8.14)
85	**53.97** **(18.22)**	-0.06 (0.24)	-10.19 (16.44)	-17.58 (21.47)	**-10.42** **(3.77)**	-4.89 (15.7)	**-10.51** **(3.77)**	-2.37 (11.07)
90	93.6 (62.48)	-0.42 (0.77)	-9.01 (9.71)	-4.97 (17.3)	-14.07 (8.25)	-12.96 (15.36)	**-10.16** **(3.76)**	-9.07 (9.87)
95	70.26 (45.5)	-0.17 (0.54)	-10.36 (8.51)	-6.55 (6.63)	-9.93 (9.07)	0.33 (24.77)	-10.11 (6.57)	-15.62 (16.76)

**Figure 1:**
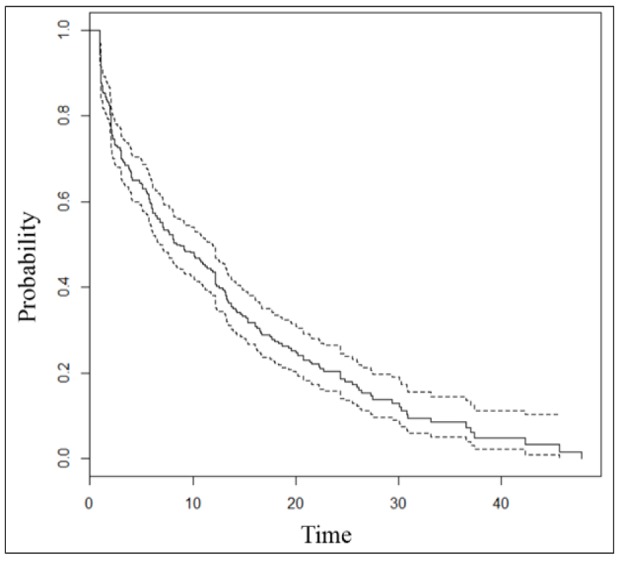
Probability of Surviving in Lung Cancer Patients

Using the estimated quantiles, it is possible to interpret Q_p_=a(*days*) so that a randomly selected person from the sample has a probability of p for experiencing the event within a days. This model uses bootstrap resampling method to estimate the coefficients’ standard error. The CQR model used the significant factors in the log-rank test results to assess the amount of adjusted effects of the independent factors on the survival of the patients. The data analysis carried out using the “survival” and “quantreg” in the statistical programing R language version 3.3.1. All statistical tests were 2-sided and a p-value<0.05 were considered statistically significant. The performance of CQR model was compared with the frequently used proportional hazards Cox regression model using the Chambless and Diao’s estimator of cumulative/dynamic AUC for right-censored time-to-event data (Chambless and Diao, 2006). To do so, the data was divided into two sets of train (70%) and test (30%) randomly. To find more reliable results, the cross-validation was repeated 500 times and the mean accuracy measure was presented for the models. The train set was utilized for model fitting and the validation of the results was checked by the test set. In order to assess the proportionality of the hazards between the levels of independent variables, the Schoenfeld residual was used. The data analysis was carried out using the “survival”, “quantreg” and “survAUC”in the statistical programing R language version 3.3.1.

## Results

The mean (± standard deviation) age of the 347 lung cancer patients was 63.48 (± 13.55) years. The survival time ranges from 11 to 91 month and the mean and median survival time was 8.73 and 5.50 months respectively. A total of 240 (67.6%) experienced death by the end of the study. Based on Figure 1 a decreasing trend of lung cancer survival probability is observed and the six-month, one, two, and three-year survival probabilities are 0.60, 0.43, 0.20, and 0.07, respectively. The patients’ characteristics are shown in Table 1. The majority of lung cancer patients were male (72.1%), group III of ECOG (45.4%), smoker (69%), NSCLC tumor (60.8%), stage IV of cancer (60%), received treatment (52.4%), and with metastasis (53.8%). Based on the estimations of the log-rank tests in Table 1, the mean survival time in the categories of ECOG, smoking habit, stage of lung cancer, receiving treatment, and metastasis (p<0.05). 

Table 2 shows the results from the CQR model. The significant variables from the unadjusted log-rank tests were entered in the adjusted analysis. The impact of selected variables in the CQR model on the survival of the patients was calculated in every five hundredth from 5th to 95th. The median survival time can be modeled as follows:

*Q(0.5 smoking, treatment receiving)*

=21.94-7.02*(if smoker)*-6.09*(if receives no treatment)*

Based on the above resulted formula, the 50^th ^percentile (median) of survival time for a smoker case who receives no treatment is 8.83 month. In other words, the probability of survival for a smoker case who receives no treatment on the 8.83th month is 50 percent. The median survival time for a non-smoker case who receives treatment increases to 21.94 month. The same formula modeling and interpretation can be implemented for any other quantiles. The 95^th^ percentile of survival time is not affected by any variables. Smoking and the status of receiving treatment are responsible for most of the survival quantiles. However, metastasis plays a significant role on the 65th survival time and ECOG (group IV) is a considerable predictor of 55^th^ and 60^th^ survival time quantiles.

## Discussion

The death risk due to lung cancer is higher for older patients than for others. In fact, with the increase in age, the death risk increases and the survival rate decreases (Zahir and Mirtalebi, 2012). But in this study, there was no significant relationship between the patient’s ages with survival time, which may be because most of the patients in this study were at a higher age.

In an evaluation of pathologic factors, the function based on ECOG system had a significant relationship with survival. Patients with different ECOG degrees had a different survival time. This variable was also significant in CQR. The lowest survival time was for patients in group IV (median survival time was 7.78 months). Thirty-fourth percent of men and 24% of women were in this group. Group III with a median survival of 13.61 months and groups I and II with a median survival of 19.22 months were in the next rank (Mitsudomi et al., 2010).

In this study, the most common type of cancer was a NSCLC tumor type, Carcinoid and SCLC were in the next rank. Some studies have also shown that the most common type of tumor is adenocarcinoma (Fitzenberger, 1997; Macdonald et al., 2004). Although the median survival rate for NSCLC was 12.32 months, the ratio of two other types of tumor (15.22 for carcinoid and 16.91 for SCLC) was lower; it did not show a significant relationship with the survival of the patients. Perhaps, the reason for this lack of communication is that most patients are at higher stages of the disease (about 89% of patients were in the third and fourth stages of the disease), which was consistent with some studies (Portnoy, 2003; Zahir and Mirtalebi, 2012).

The tumor stage showed a significant relationship with survival time. People at higher risk of death had a higher mortality rate than other patients. Patients with a tumor stage of IV type had a median survival of 11.66 years and those with other stages of the tumor (I, II, and III) with a median survival of 15.88 months. This was consistent with the study by Kawaguchi et al., (2010).

In this study, people who experienced at least one of the treatment ways, such as chemotherapy, radiotherapy or surgery were compared with those who did not have any treatment or surgery. The survival of the two groups was significantly different. A group experiencing at least one type of treatment or surgery had a median survival of 14.77 months and the non-treated group had a mean survival of 6.6 months. This was consistent with some studies (Chambless and Diao, 2006; Zahir and Mirtalebi, 2012). The results of this study showed that smoking was the most important cause of death in patients with lung cancer because smokers had a lower survival rate compared to non-smokers (11.36 months for smokers and 16.02 months for non-smokers). In fact, lung cancer has a high correlation with the patients’ smoking status. In studies conducted by Mitsudomi et al., (2010) and Kawaguchi et al., (2010), smoking or non-smoking is a variable influencing the survival time.

Lung metastasis was another effective variable in survival time. Patients with lung metastasis showed a median survival of 10.47 months, and patients who did not have metastasis showed a median survival time of 15.76 months.

Moreover, we used censored quantile regression to find the probability of survival in any desired quantile. Regarding the skewness of time-to-event data, semi-parametric approaches such as quantile regression models can fit the data better and result in more valid estimations and interpretations (Portnoy, 2003). 

There were some limitations about the relatively small sample size in our data. Moreover, missing data was frequently observed in the patients’ records in hospital and health centers and information about some other potential risk factors have not been measured.

It has been shown that male, no surgery, longer time between symptom and diagnosis, low scores of socioeconomic status and older age are significant risk factors in reducing the probability of survival from esophageal cancer. And we can conclude that Eastern Cooperative Oncology, smoking habit and treatment were significantly associated with a shorter time-to-death progression. As censored quintile regression could consider time-varying effects and the interpretation of its regression coefficients are easy, it could be an adequate choice for analyzing survival data.
